# Supplementary figures and images for: Multi-clonal SARS-CoV-2 neutralization by antibodies isolated from severe COVID-19 convalescent donors
Source: PLoS Pathog. 2021 Feb 11;17(2):e1009165. doi: 10.1371/journal.ppat.1009165 (PMC7877634; doi:10.1371/journal.ppat.1009165)

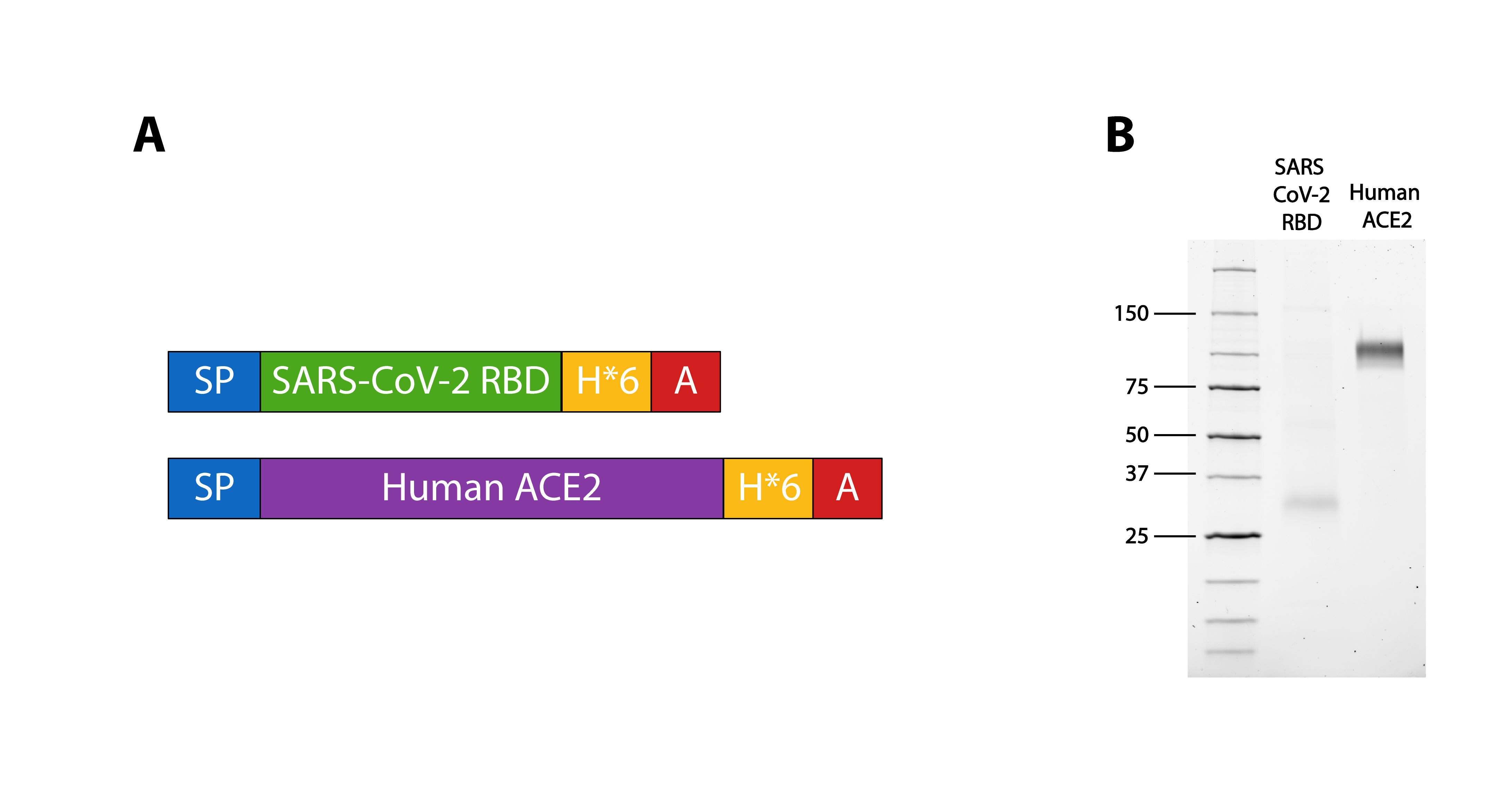

Supplement: S1 Fig — (A) Schematic illustration of the SARS-CoV-2 RBD (top) and human ACE2 (bottom) constructs that were cloned into the pcDNA 3.1 vector. Each construct contains a human secretion signal at the 5’ (SP, indicated in blue) and two tags at the 3’ end; a hexa histidine tag (H*6, indicated in yellow) and an AviTag (“A”, indicated in red). (B) Representative protein gel image of the purified SARS-CoV-2 RBD (middle lane) and human ACE2 protein (right lane). The standard protein ladder is shown in the left lane. (TIF) [file ppat.1009165.s001.tif]

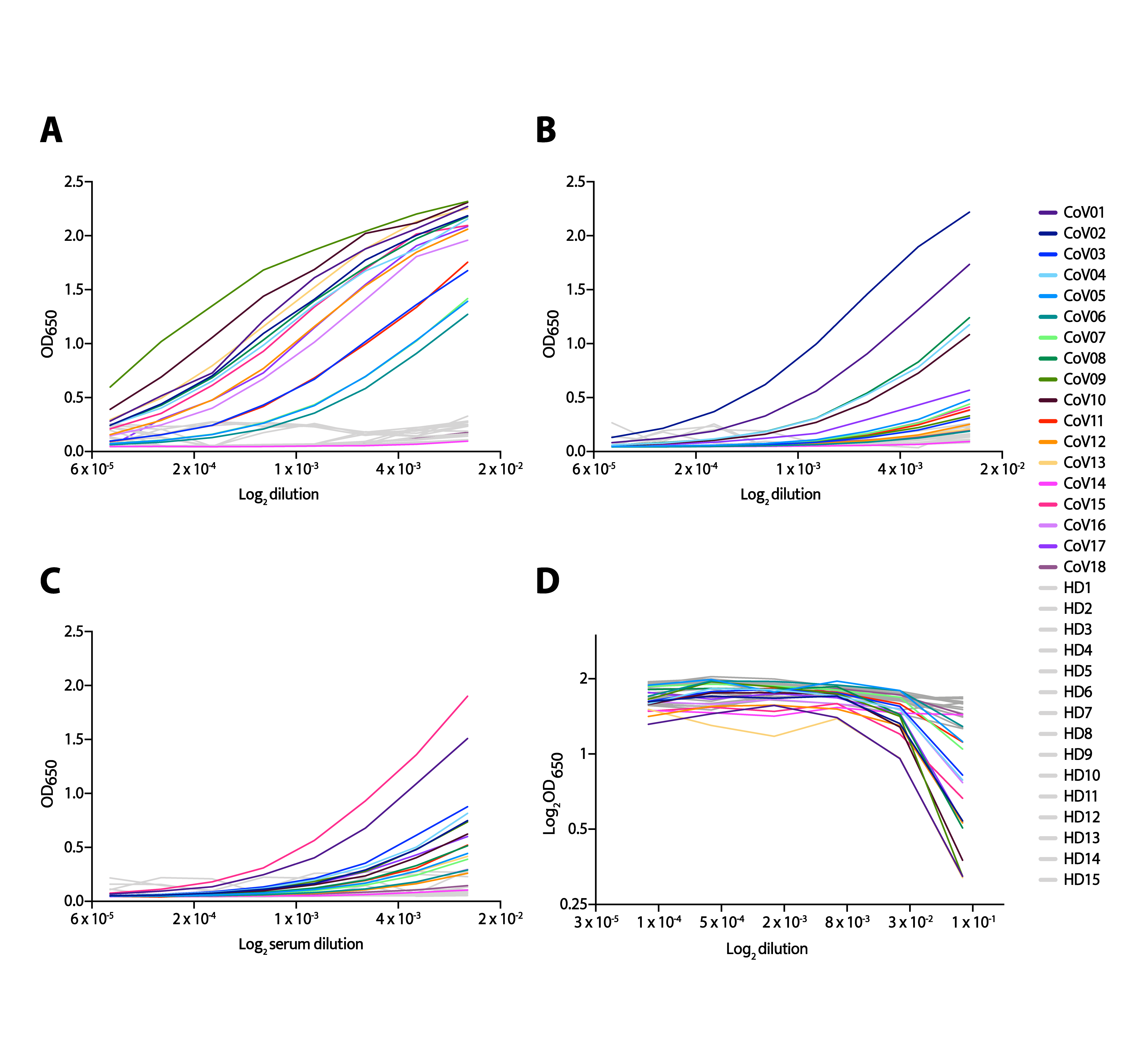

Supplement: S2 Fig — (A-C) IgG, IgM and IgA plasma responses of 18 SARS-CoV-2 convalescent donors (CoV01-CoV18) against the SARS-CoV-2 RBD protein. Plasma was diluted 1:100 with 7 additional consecutive 2-fold dilutions. The color-code for each donor is indicated on the right of (B). (D) Patient plasma inhibition of RBD:ACE2 binding in ELISA. Plasma was diluted 1:10 with 5 additional consecutive 4-fold dilutions. Lower OD correlates to higher inhibition. The x-axes are represented as log2 of the dilutions. The y-axis is represented as log2 of the OD650 values. (TIF) [file ppat.1009165.s002.tif]

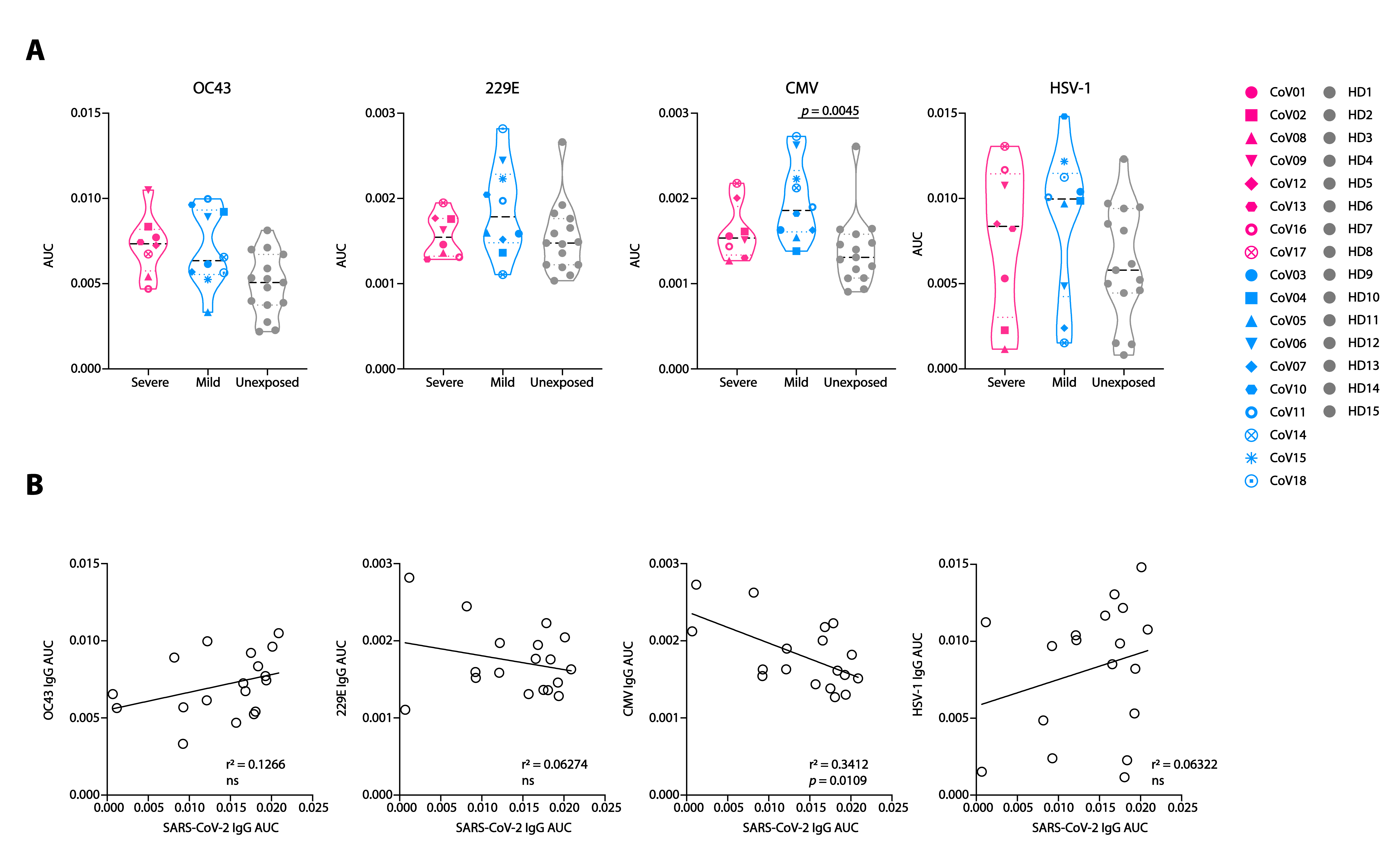

Supplement: S3 Fig — (A) Area under the curve (AUC) values for IgG binding from 18 SARS-CoV-2 convalescent donors (CoV01-CoV18) to OC43, 229E, CMV or HSV-1 (viral particles). (B) Correlation between anti-SARS-CoV-2 IgG serum reactivity (AUC, Fig 1A) to IgG AUC against OC43, 229E, CMV or HSV-1. AUC and correlations were calculated using GraphPad Prism software. Statistical analysis was performed using one-way ANOVA test. (TIF) [file ppat.1009165.s003.tif]

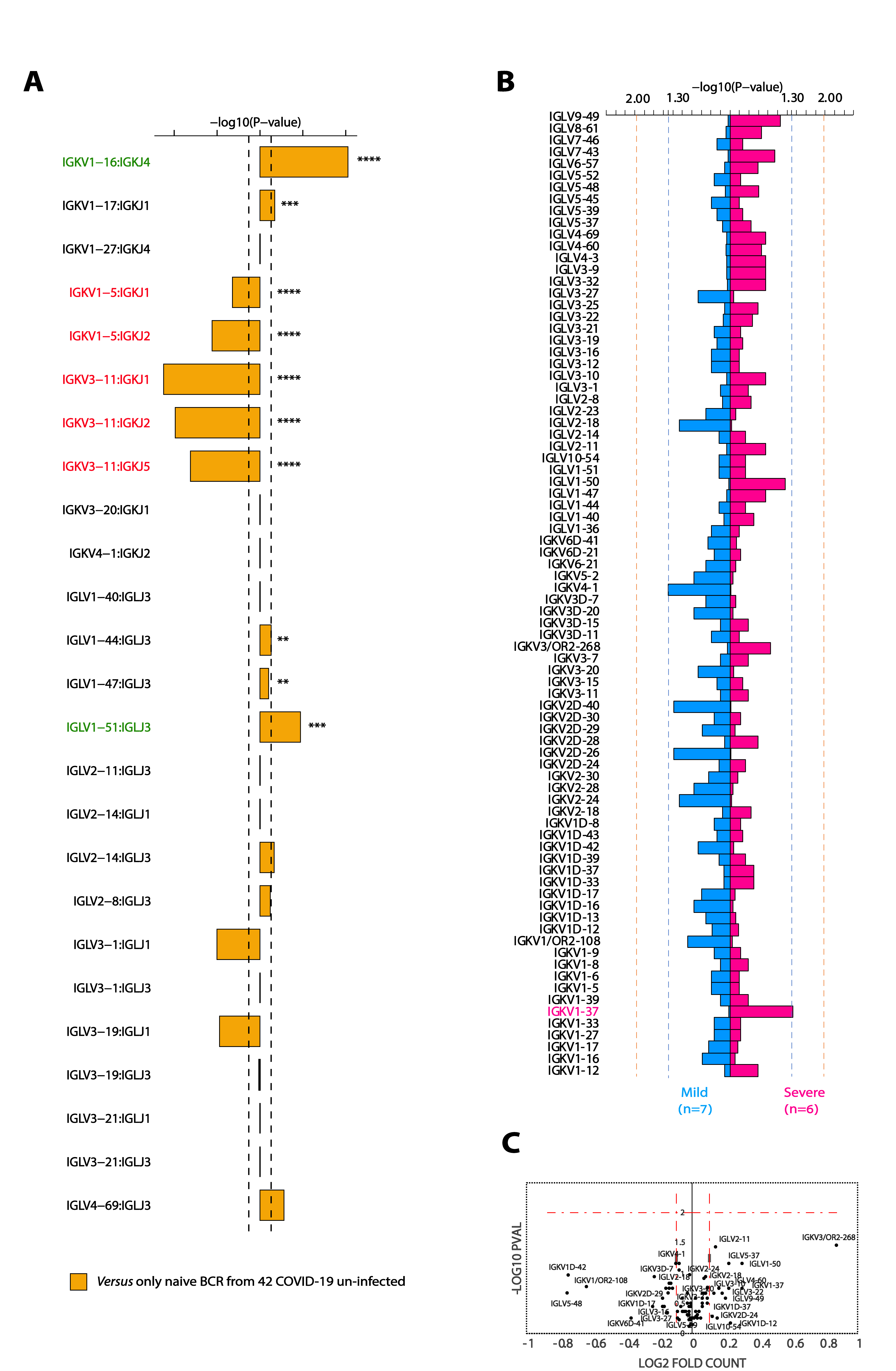

Supplement: S4 Fig — (a) Frequencies of the top 25 VLJL combinations in BCRs from 13 COVID-19 donors versus BCRs from 42 healthy individuals naïve B cells (orange bars). -Log10 p values were calculated using Mann Whitney test with FDR correction. Over-represented VHJH combinations are marked in green, while under-represented are marked in red. p values are listed by the corresponding horizontal column. (b) Comparison between the frequencies of 82 VL genes in Severe versus Mild COVID-19 donors. Magenta right pointing horizontal bars indicate VL genes that are over-represented in Severe donors over Mild, while light blue left pointing horizontal bars indicate VL genes that are over-represented in Mild donors over Severe. Blue dashed lines correspond to p < 0.05 and red dashed lines corresponds to p < 0.01. p values were calculated using Mann Whitney test. (c) Volcano plot showing p value as calculated in (b) and normalized fold counts for every segment. ** p < 0.01 *** p < 0.001 **** p < 0.0001. (TIF) [file ppat.1009165.s004.tif]

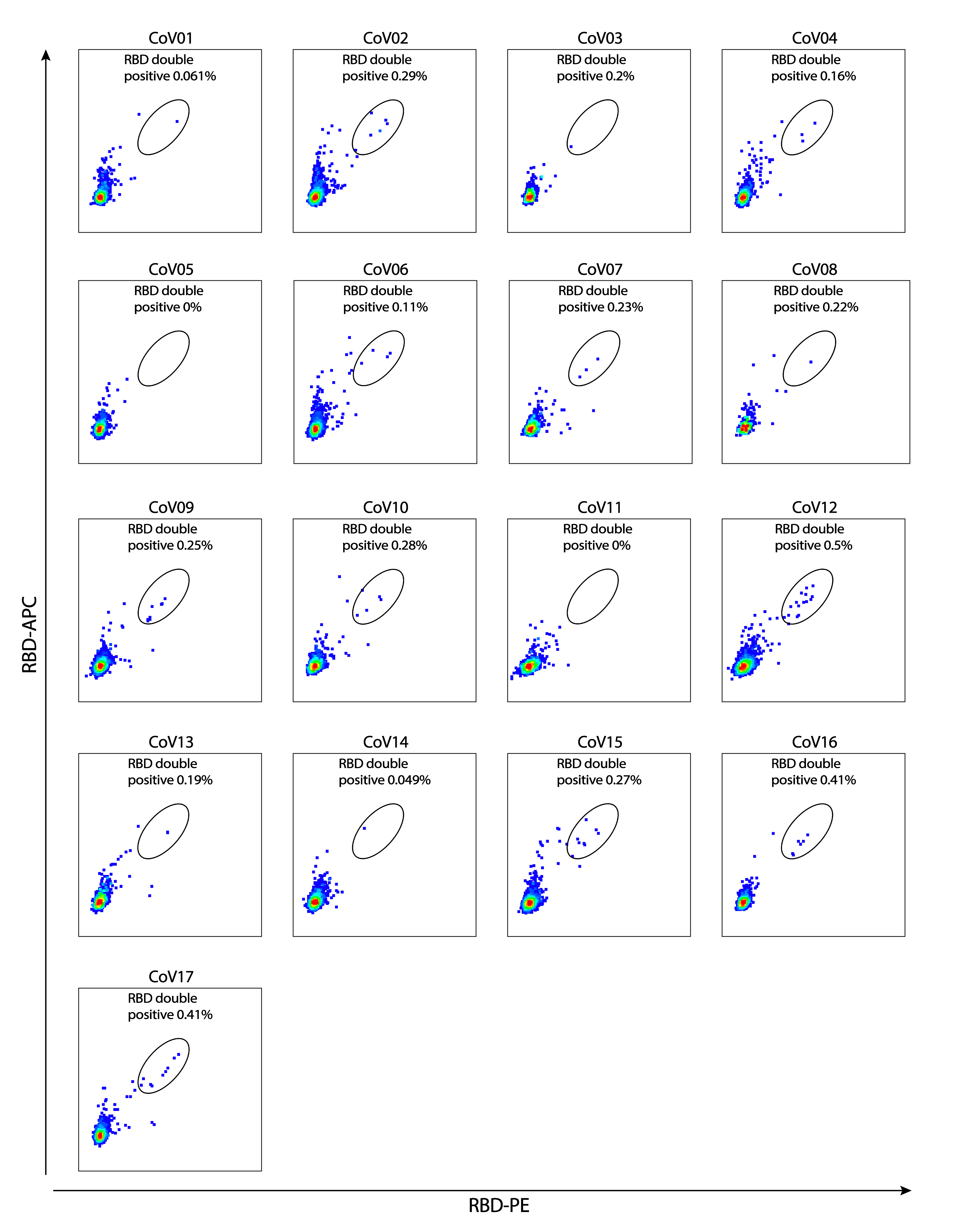

Supplement: S5 Fig — Flow cytometry plots showing APC-RBD and PE-RBD double stained memory B cells for CoV01-17 (no PBMCs were obtained for CoV18). The frequencies of double positive B cells are indicated within each plot. (TIF) [file ppat.1009165.s005.tif]

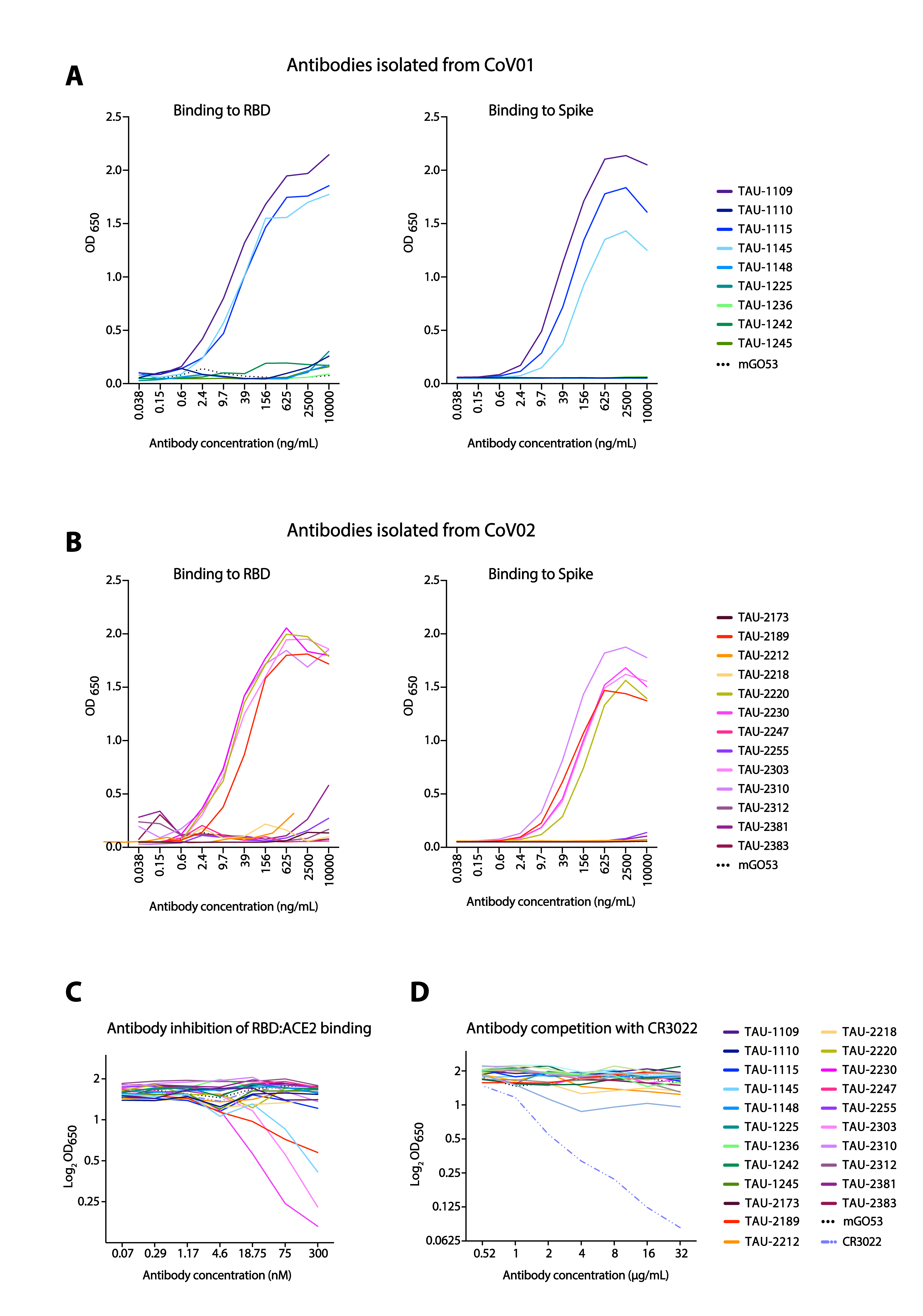

Supplement: S6 Fig — (A) and (B) CoV01 and CoV02 mAbs, respectively, binding to SARS-CoV-2 RBD (left) and Spike trimer (right). The color-code is indicated to the right of each graph. (C) Antibody inhibition of RBD:ACE2 binding in ELISA. Antibodies were assayed at 300 nM with 6 additional consecutive 4-fold dilutions. The y-axis is represented as log2 of the OD650 values. Lower OD indicates higher mAb inhibition. (D) Antibody competition with biotinylated-CR3022. Lower OD650 values indicate a higher level of competition between the mAbs. (TIF) [file ppat.1009165.s006.tif]

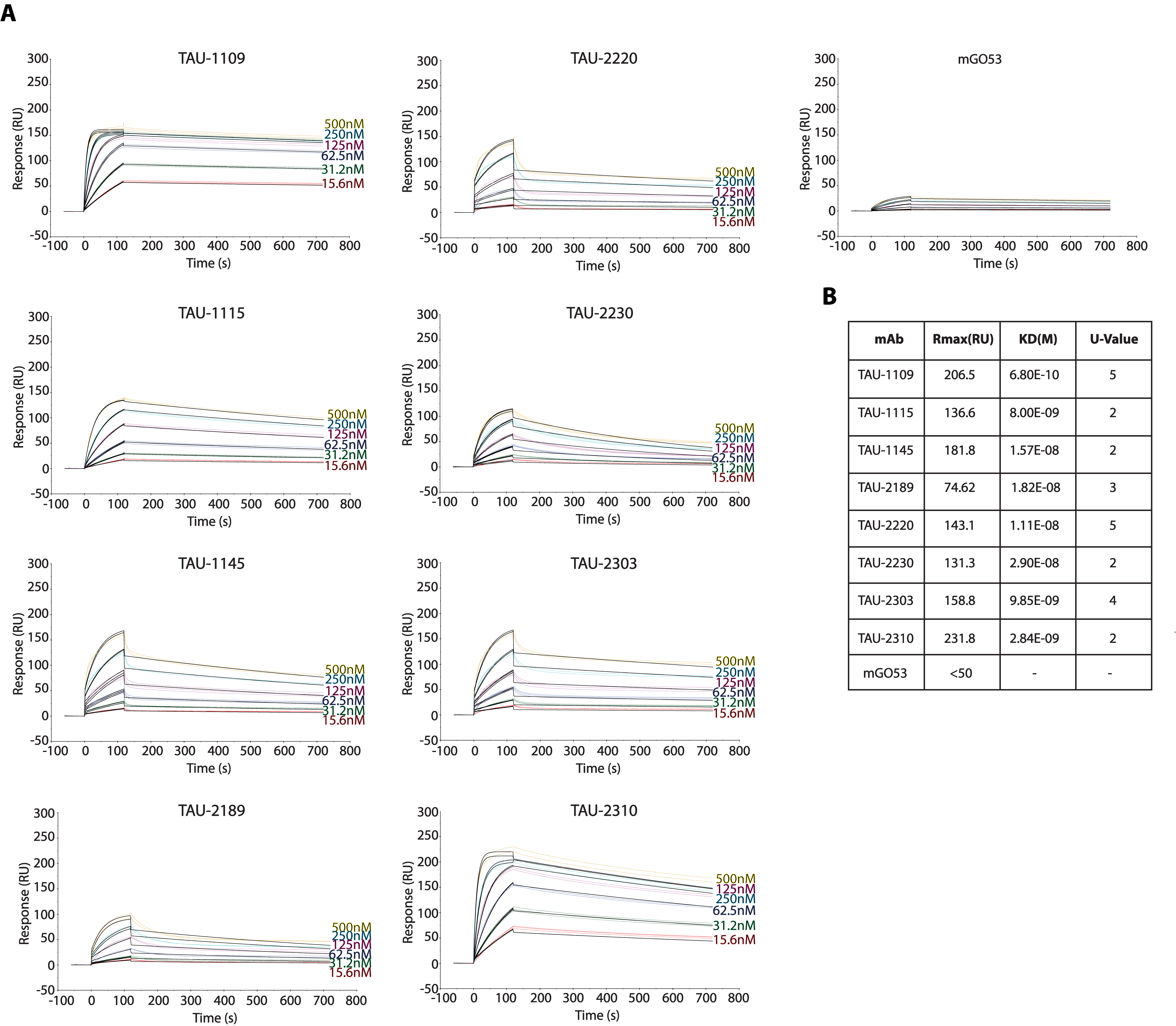

Supplement: S7 Fig — (A) SPR sensograms showing binding of injected SARS-CoV-2 RBD at six different concentrations (15.6 nM, 31.25 nM, 62.5 nM, 125 nM, 250 nM and 500 nM) to immobilized anti-SARS-CoV-2 TAU mAbs (0.5 μg/ml). mGO53 was used as isotype control. SPR assays were performed on a Biacore T200 instrument at 25°C. Three replicates were performed for each mAb and all samples were diluted in HBS-EP buffer (0.01 M HEPES, 0.15 M NaCl, 0.003 M EDTA, 0.05% Tween 20, pH 7.4). Sensograms were fitted to 1:1 binding model using non-linear regression in the biaevaluation software. KD was calculated using the ratio of the kinetic constants KD = Kd/Ka. (B) Rmax, KD, and U-value values. (TIF) [file ppat.1009165.s007.tif]

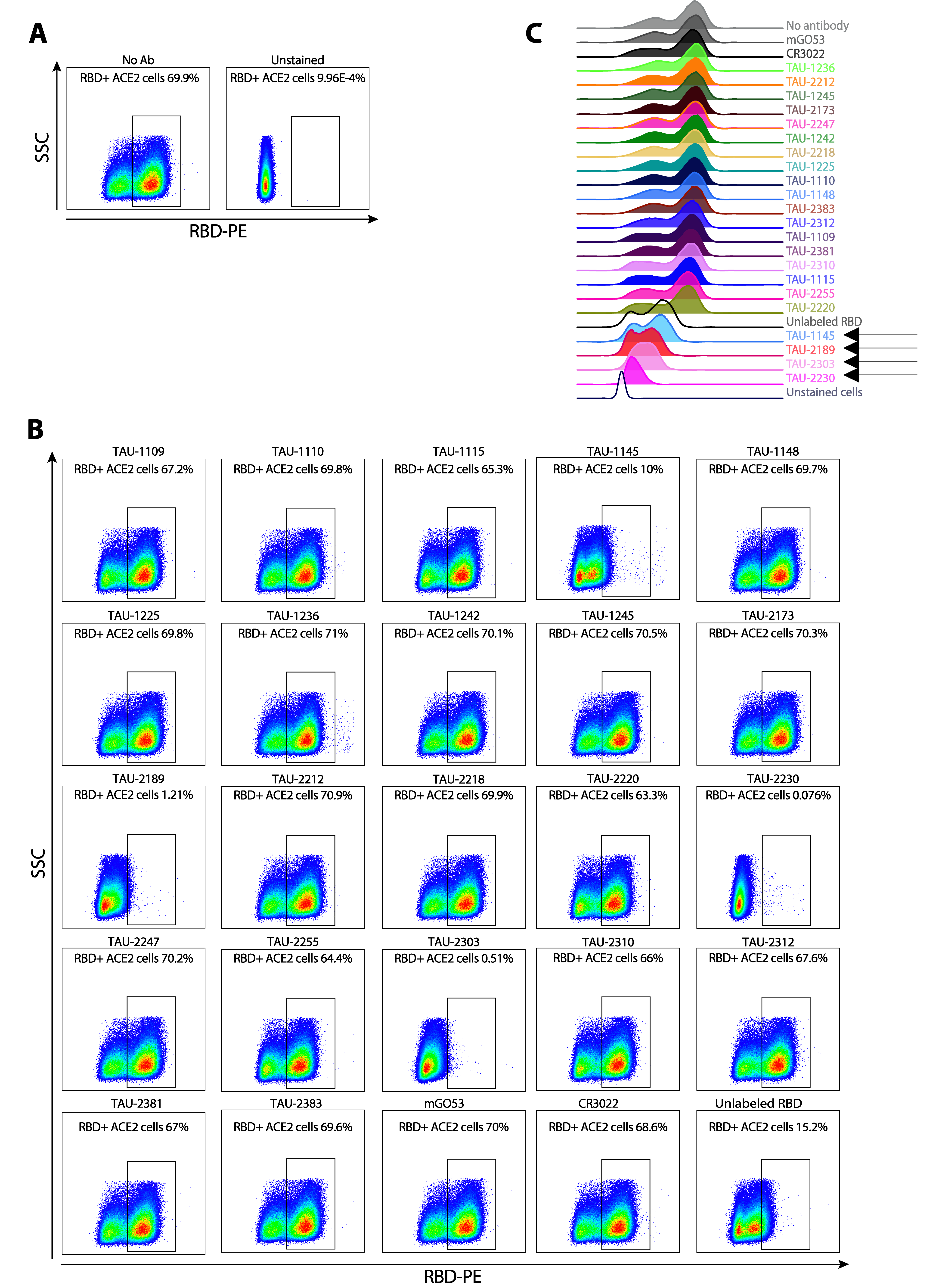

Supplement: S8 Fig — (A) Flow cytometry plots showing hACE2-expressing cells stained with RBD-PE (“no Ab”, left) and unstained hACE2-expressing cells (right). (B) Anti-SARS-CoV-2 mAbs were pre-incubated with RBD-PE followed by incubation with hACE2-expressing cells. Unlabeled RBD was used as a positive control, and mGO53 as a negative control [42]. The frequencies of PE positive cells are indicated. (C) Mean fluorescence intensity (MFI) of RBD-PE stained hACE2-expressing cells identified by flow cytometry in the presence of mAbs (mAbs that reduced RBD-PE staining are marked with black arrows). (TIF) [file ppat.1009165.s008.tif]

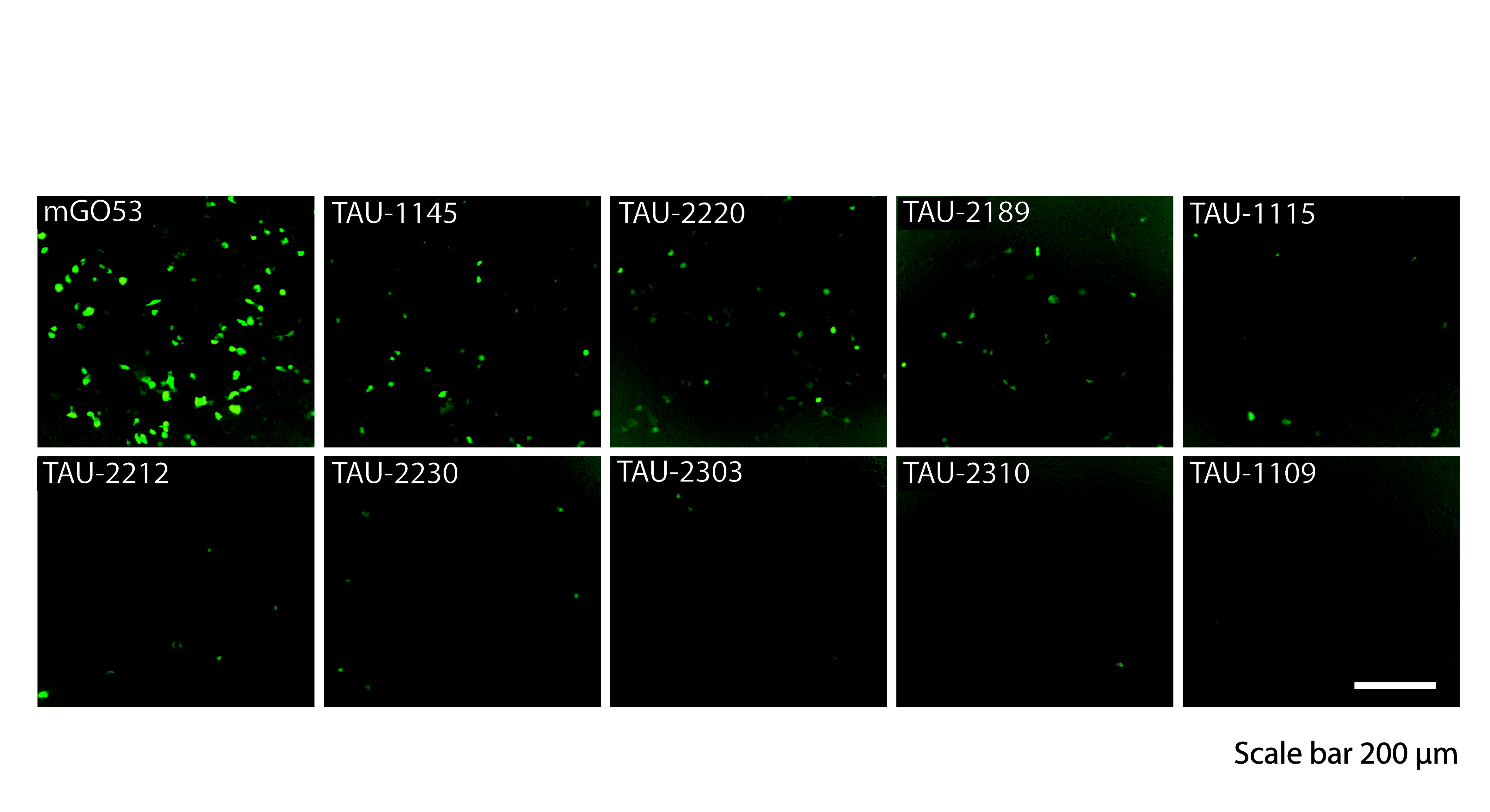

Supplement: S9 Fig — Representative images of HEK-293 cells stably expressing hACE2 infected with SARS-CoV-2-Spike GFP-expressing pseudo-particles in the presence of mAbs TAU-1145, -2220, -2189, -1115, -2212, -2230, -2303, -2310, and -1109, as well as the negative control mAb mGO53 [42]. Cells were imaged 24 h post infection using IncuCyte ZOOM. (TIF) [file ppat.1009165.s009.tif]

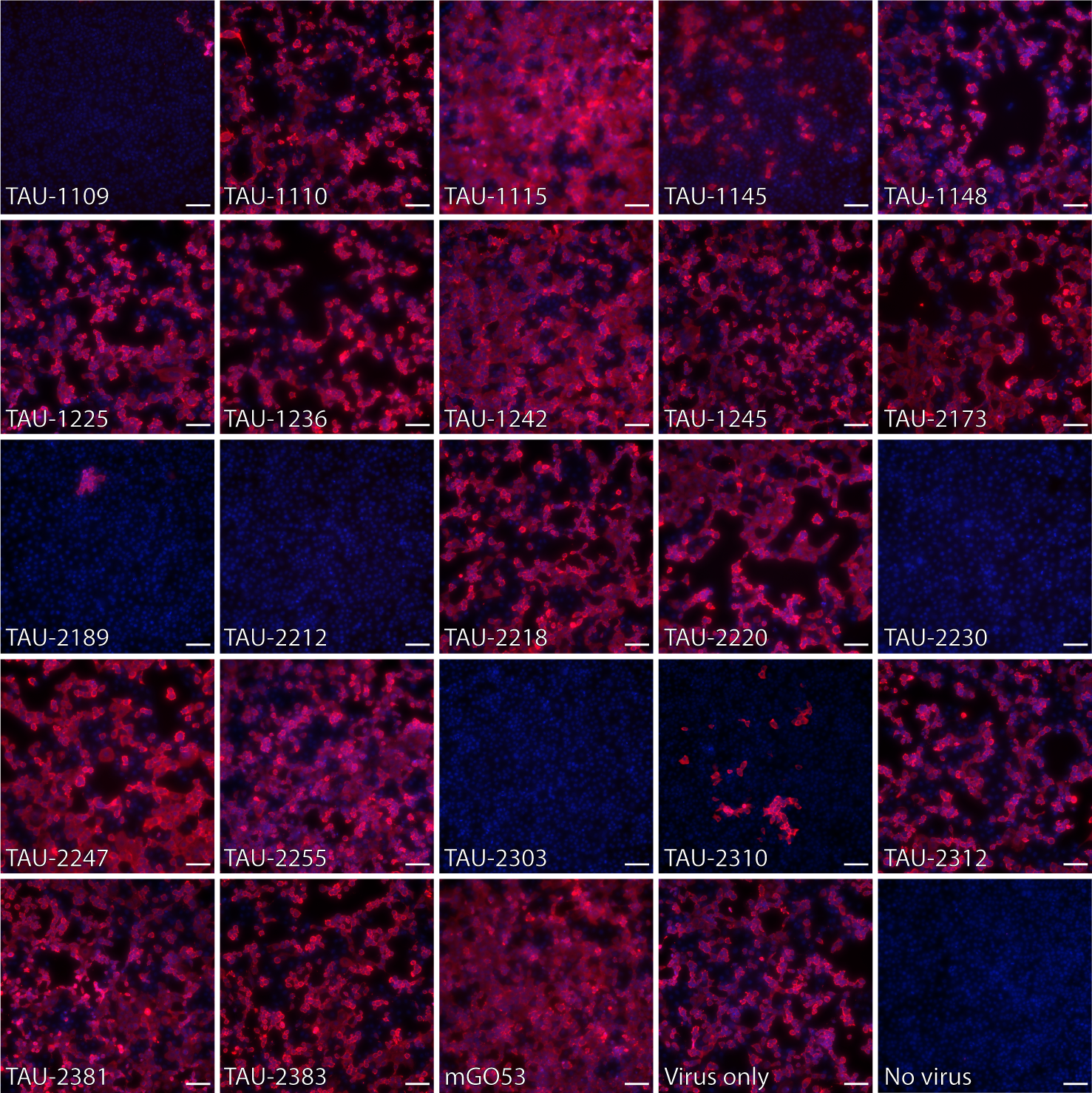

Supplement: S10 Fig — Images of Vero E6 cells infected with authentic SARS-CoV-2 following fixation and staining with nucleocapsid antibody AF594 and Hoechst nuclear staining. Virus and antibodies were pre-incubated for 1 h prior to infection. mGO53 is shown as a human isotype control [42]. (TIF) [file ppat.1009165.s010.tif]

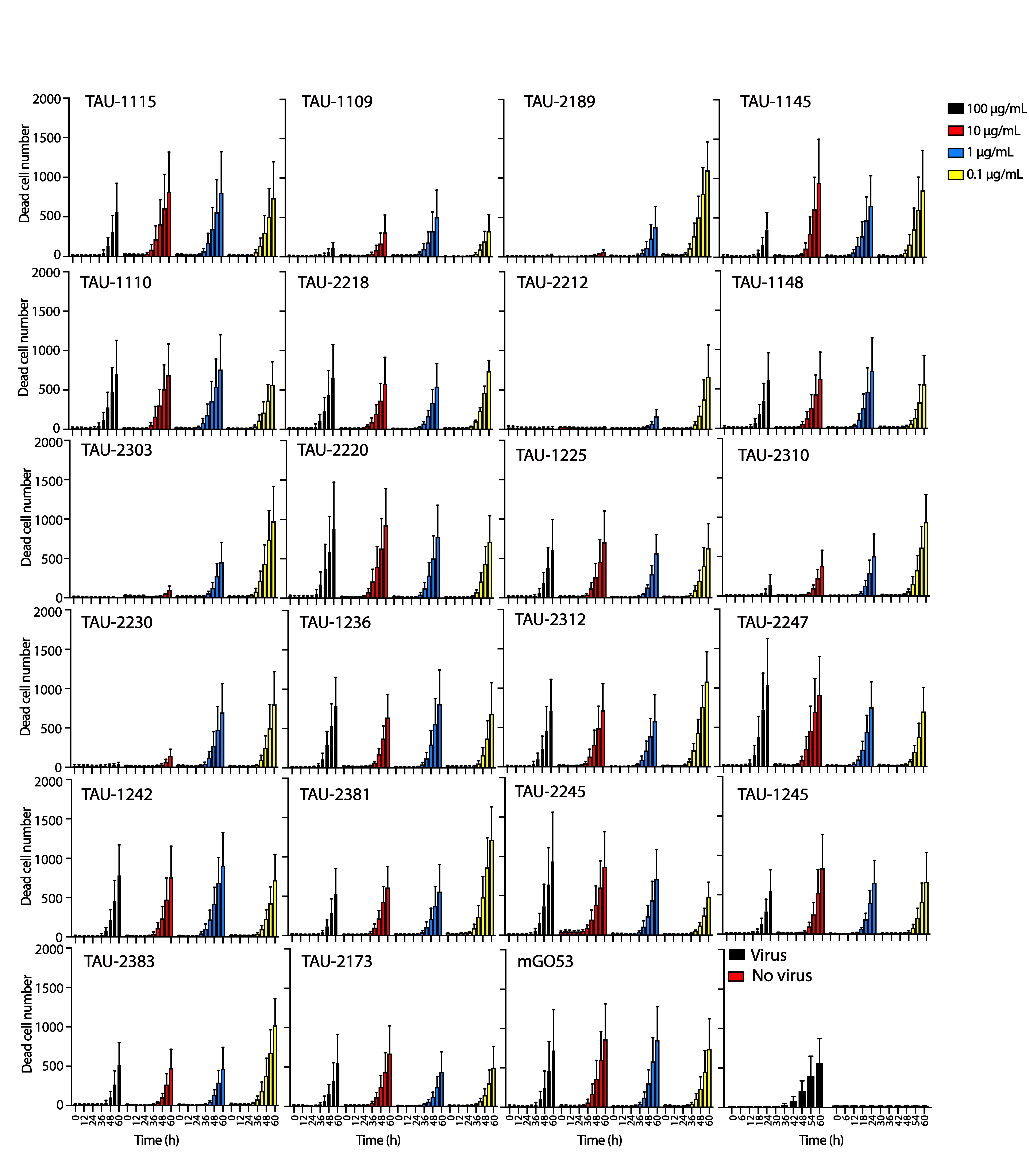

Supplement: S11 Fig — Confluent Vero E6 cells were infected with SARS-CoV-2 at MOI:1 in the presence of a titration of the 22 mAbs along with controls. Four different concentrations used are labeled black (100 μg/mL), red (10 μg/mL), blue (1 μg/mL), and yellow (0.1 μg/mL). Viability of cells in five fields of view was monitored with propidium iodide every 6 h for 60 h using an Incucyte S3. (TIF) [file ppat.1009165.s011.tif]

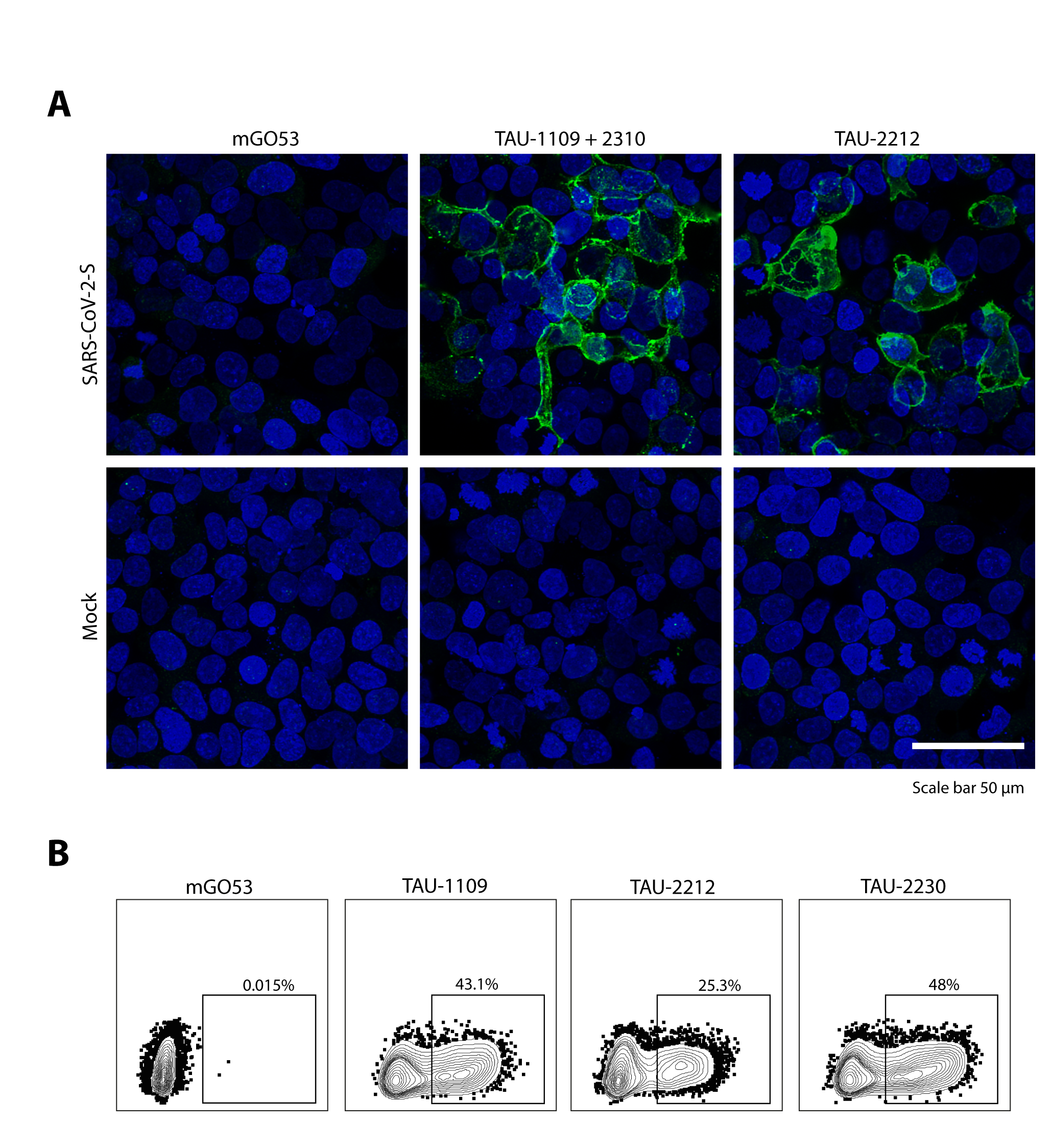

Supplement: S12 Fig — (A) Confocal microscopy images of HEK-293 cells transiently expressing SARS-CoV-2-Spike (MN908947.3) and stained with TAU-2212 and a mix of TAU-1109 and TAU-2230 followed by incubation with FITC-conjugated anti-human secondary antibody. mGO53 serves as an isotype control [42]. (B) Flow cytometry plots of Expi293F cells transiently expressing SARS-CoV-2-Spike incubated with TAU-1109, TAU-2212 and TAU-2230 and stained with APC-conjugated anti-human secondary antibody. mGO53 serves as an isotype control [42]. (TIF) [file ppat.1009165.s012.tif]

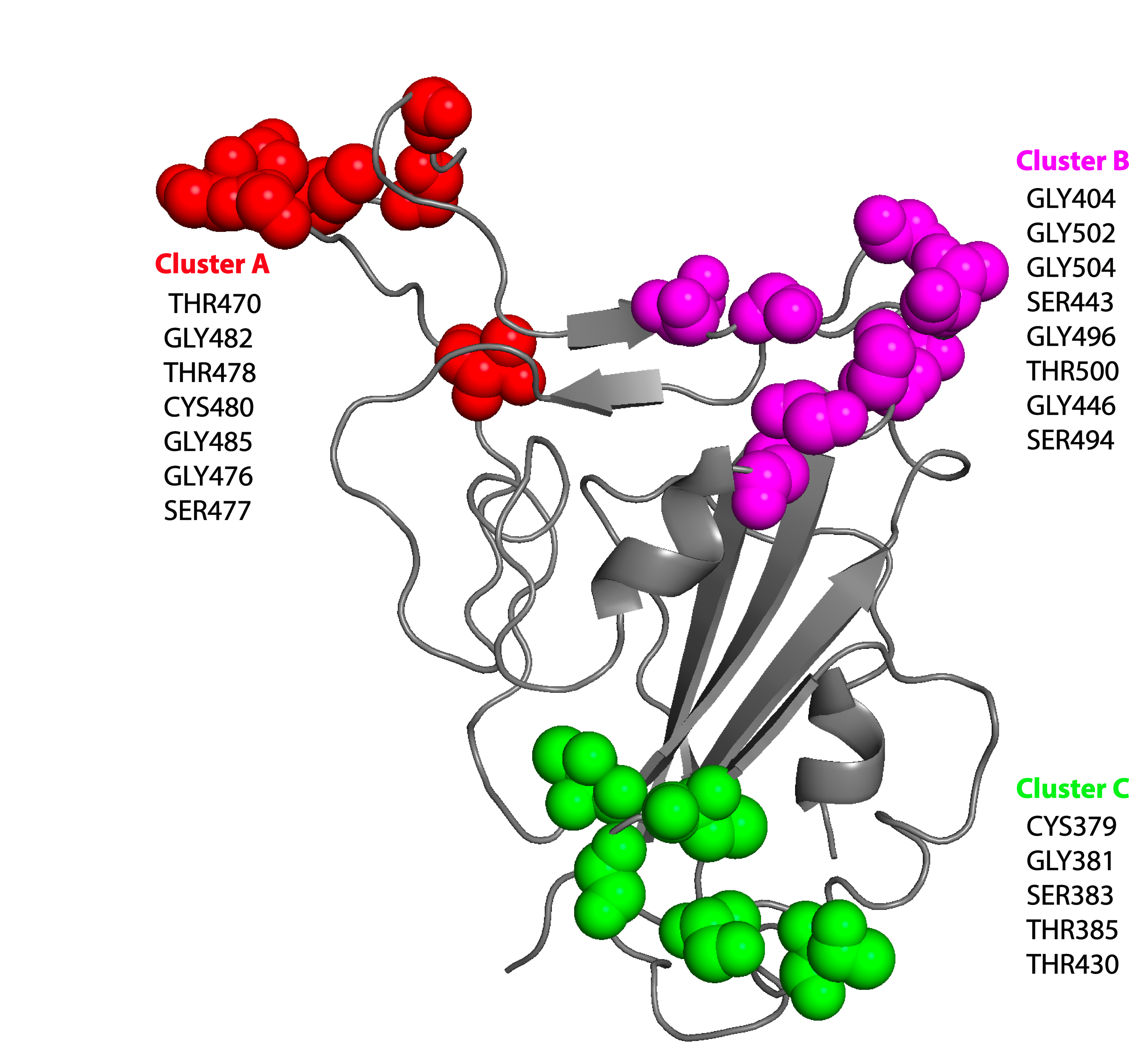

Supplement: S13 Fig — Three predicted epitope-candidates, Clusters A, B and C are depicted in red, magenta and green, respectively. The predicted residues are shown in spacefill on the surface of SARS-CoV-2 RBD (PDB ID 6M0J [4]). The amino acid residues comprising each cluster are listed. The prediction was conducted using the D = 9 A, ST = 6, f = 0 parameters. (TIF) [file ppat.1009165.s013.tif]

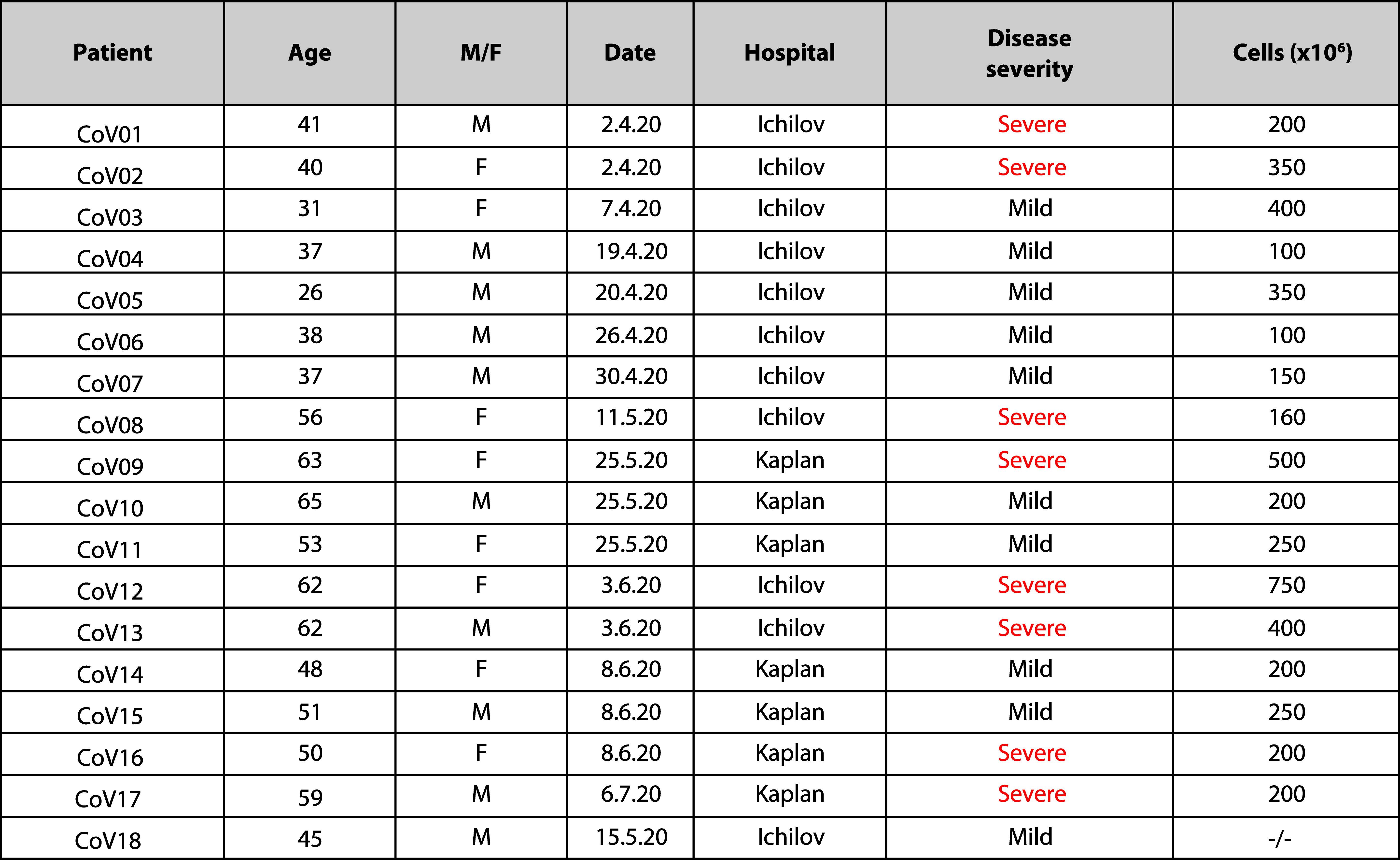

Supplement: S1 Table — (TIF) [file ppat.1009165.s014.tif]

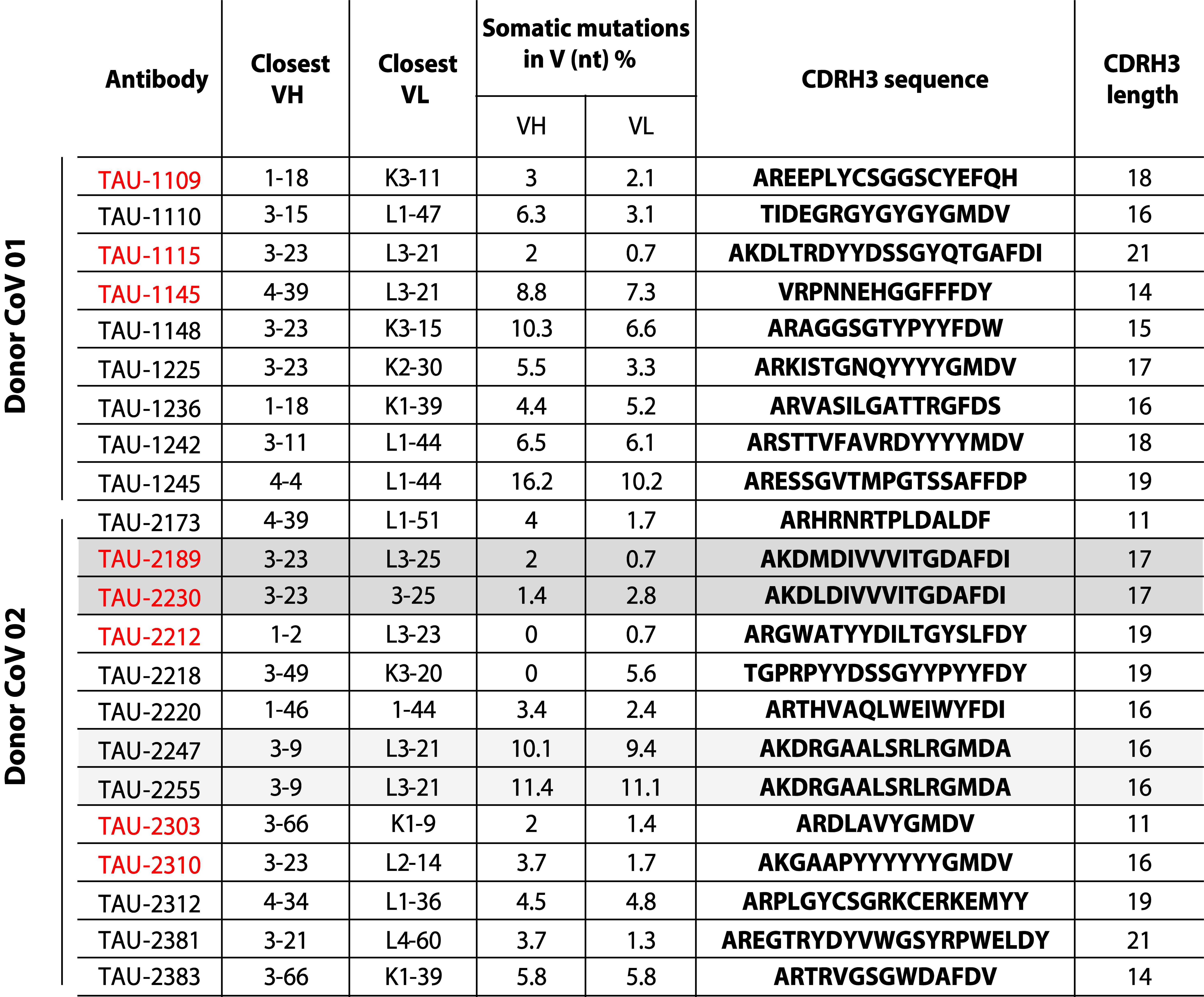

Supplement: S2 Table — The donor from whom each mAb was isolated is indicated to the left. Neutralizing mAbs are colored in red. Clonal relatives are shaded in the same color. (TIF) [file ppat.1009165.s015.tif]
