# Supplementary material for: Multi-clonal SARS-CoV-2 neutralization by antibodies isolated from severe COVID-19 convalescent donors
Source: PLoS Pathog. 2021 Feb 11;17(2):e1009165. doi: 10.1371/journal.ppat.1009165 (PMC7877634; doi:10.1371/journal.ppat.1009165)
Supplement: Attachment — Submitted filename: Response_to Reviewers.docx [file ppat.1009165.s016.docx]

Response to the Reviewers:

Color code:

*Reviewer’s text*

Author’s response

Manuscript text

Changes in the text

**Reviewer #1**

We thank the reviewer for pointing out that *“, the experiments and analyses are of very high quality and importance, carefully reported, and important to the field. Rigorous methodology was applied*.”

**Reviewer #1: “Major issues”:**

*1. Antibodies are made of heavy and light chains (H and L). Particular VHJH sequences were found to be enriched in COVID patients. The same analysis should be performed for the light chains, and if possible for the heavy and light chains combined.*

We thank the reviewer for this valuable comment. In order to address it, we performed all the analyses on the light chains, including it in a new **Supplementary Figure 4**, with the exception of the mature Covid-19-uninfected database, where we only had the heavy chain BCR sequences and not the light chain BCR sequences. Additionally, as requested, we included the analysis of the paired heavy and light chains as a new panel in the main **Figure 2f**.

The following changes were made in the text (Page 5):

“

When analyzing the light chains, COVID-19 samples had enriched representation of two VKJK combinations, while five VKJK combinations were under-represented when compared to 42 BCR repertoires from naïve B cells (Supplementary Figure 4a). Next, we compared the BCR sequences between Mild and Severe COVID-19 donors. Mild COVID-19 donors had significantly lower B cell lineage overlap compared to the B cell lineage overlap within the Severe group (55 shared B cell clones in Mild, compared to 96 shared B cell clones in Severe, Figure 2c). In VH3-53 gene was significantly over-expressed in the Severe group when compared to Mild (*p* = 0.004, Figure 2d and e), whereas no significant differences in VK gene usage was found between the two groups of CODIV-19 donors (Supplementary Figure 4b and c). When comparing both heavy and light chains, 18 VHVL combinations were enriched in Severe, and 4 VHVL combinations were enriched in the Mild patients. Overall these results indicate that severe SARS-CoV-2 infection is associated with unique B cell signatures, enrichment of VH3-53, and a greater B cell clonal expansion.

“

This is the new addition to the legend of Figure 2 (Page 12):

(**f**) Comparison between the frequencies of 2500 VH-VL pairs in COVID-19 in Severe *versus* Mild groups of donors. Only pairs that are below p < 0.01 are indicated. Color code, configuration and *p* values are the same as in (**e**). ** *p* < 0.01 *** *p* < 0.001 **** *p* < 0.0001.

This is the legend of the new Supplementary Figure 4 (Pages 26-27):

**Supplementary Figure 4: Frequency of light chains in COVID-19 convalescent donors.**

Frequencies of the top 25 VLJL combinations in BCRs from 13 COVID-19 donors *versus* BCRs from 42 healthy individuals naïve B cells (orange bars). -Log_10_ *p* values were calculated using Mann Whitney test with FDR correction. Over-represented VHJH combinations are marked in green, while under-represented are marked in red. *P* values are listed by the corresponding horizontal column. (**b**) Comparison between the frequencies of 82 VL genes in Severe *versus* Mild COVID-19 donors. Magenta right pointing horizontal bars indicate VL genes that are over-represented in Severe donors over Mild, while light blue left pointing horizontal bars indicate VL genes that are over-represented in Mild donors over Severe. Blue dashed lines correspond to *p* < 0.05 and red dashed lines corresponds to *p* < 0.01. *p* values were calculated using Mann Whitney test. (**c**) Volcano plot showing *p* value as calculated in (**b**) and normalized fold counts for every segment. ** p < 0.01 *** p < 0.001 **** p < 0.0001.

*2. It is stated that ‘data are available with no restrictions’ but could not find table with all antibody sequences related to Figure 2, and the full sequence of the cloned monoclonal antibodies is missing. This information should be included.*

# The sequences related to Figure 2 were uploaded to SRA (Sequence Read Archive) with the submission number SUB8326459. The full cloned antibody sequences will be deposited to NCBI database and will be fully available once the manuscript is published.

# Accession Sample Name SPUID Organism Tax ID Isolate

# SAMN16513410 COV1 COV1 Homo sapiens 9606 COV1

# SAMN16513411 COV2 COV2 Homo sapiens 9606 COV2

# SAMN16513412 COV3 COV3 Homo sapiens 9606 COV3

# SAMN16513413 COV4 COV4 Homo sapiens 9606 COV4

# SAMN16513414 COV5 COV5 Homo sapiens 9606 COV5

# SAMN16513415 COV6 COV6 Homo sapiens 9606 COV6

# SAMN16513416 COV7 COV7 Homo sapiens 9606 COV7

# SAMN16513417 Contact3 Contact3 Homo sapiens 9606 Contact3

# SAMN16513418 CoV9 CoV9 Homo sapiens 9606 CoV9

# SAMN16513419 Cov10 Cov10 Homo sapiens 9606 Cov10

# SAMN16513420 Cov12 Cov12 Homo sapiens 9606 Cov12

# SAMN16513421 Cov13 Cov13 Homo sapiens 9606 Cov13

# SAMN16513422 Cov14 Cov14 Homo sapiens 9606 Cov14

# SAMN16513423 Cov15 Cov15 Homo sapiens 9606 Cov15

# SAMN16513424 Cov16 Cov16 Homo sapiens 9606 Cov16

# Object IDs and corresponding URLs:

# 16513410: https://www.ncbi.nlm.nih.gov/sra/16513410

# 16513411: https://www.ncbi.nlm.nih.gov/sra/16513411

# 16513412: https://www.ncbi.nlm.nih.gov/sra/16513412

# 16513413: https://www.ncbi.nlm.nih.gov/sra/16513413

# 16513414: https://www.ncbi.nlm.nih.gov/sra/16513414

# 16513415: https://www.ncbi.nlm.nih.gov/sra/16513415

# 16513416: https://www.ncbi.nlm.nih.gov/sra/16513416

# 16513417: https://www.ncbi.nlm.nih.gov/sra/16513417

# 16513418: https://www.ncbi.nlm.nih.gov/sra/16513418

# 16513419: https://www.ncbi.nlm.nih.gov/sra/16513419

# 16513420: https://www.ncbi.nlm.nih.gov/sra/16513420

# 16513421: https://www.ncbi.nlm.nih.gov/sra/16513421

# 16513422: https://www.ncbi.nlm.nih.gov/sra/16513422

# 16513423: https://www.ncbi.nlm.nih.gov/sra/16513423

# 16513424: https://www.ncbi.nlm.nih.gov/sra/16513424

# 16513410: https://www.ncbi.nlm.nih.gov/sra/16513410

# 16513411: https://www.ncbi.nlm.nih.gov/sra/16513411

# 16513412: https://www.ncbi.nlm.nih.gov/sra/16513412

# 16513413: https://www.ncbi.nlm.nih.gov/sra/16513413

# 16513414: https://www.ncbi.nlm.nih.gov/sra/16513414

# 16513415: https://www.ncbi.nlm.nih.gov/sra/16513415

# 16513416: https://www.ncbi.nlm.nih.gov/sra/16513416

# 16513417: https://www.ncbi.nlm.nih.gov/sra/16513417

# 16513418: https://www.ncbi.nlm.nih.gov/sra/16513418

# 16513419: https://www.ncbi.nlm.nih.gov/sra/16513419

# 16513420: https://www.ncbi.nlm.nih.gov/sra/16513420

# 16513421: https://www.ncbi.nlm.nih.gov/sra/16513421

# 16513422: https://www.ncbi.nlm.nih.gov/sra/16513422

# 16513423: https://www.ncbi.nlm.nih.gov/sra/16513423

# 16513424: https://www.ncbi.nlm.nih.gov/sra/16513424

**Reviewer #1 “Minor issues”:**

*1. ‘live SARS-CoV-2’: presumably the authors mean ‘authentic SARS-CoV-2’ as opposed to pseudo virus, consider re-wording*

We thank the reviewer for this comment, we changed the text accordingly 14 times throughout the text and Figure legends:

- Abstract page 2:” neutralization against authentic SARS-CoV-2, and inhibited syncytia formation Introduction page 4: efficacy alone and in combination against authentic SARS-CoV-2.”
- Results page 6:”We then tested the neutralizing activity of the mAbs against authentic SARS-CoV-2. Vero E6 cells were infected with authentic SARS-CoV-2 in the presence of mAbs, and after 48 h virus was detected using fluorescently labeled anti-nucleocapsid antibody. “
- Results page 7:”Six of the nine mAbs that neutralized the pseudo-typed virus, also neutralized authentic virus (TAU-1109, -2212, -2189, -2230, -2310, and -2303, Figure 4b and c, Supplementary Figure 8). “
- Results page 7:”Interestingly, mAb TAU-2212 demonstrated impressive neutralizing ability in all three assays (pseudo-virus, authentic virus and syncytia formation) “
- Results page 8:tracked by authentic cell imaging for three days to examine the effects of mAb titrations on virus-induced cell death.”
- Results page 8:”…blocked authentic SARS-CoV-2 infection by 95%,..”
- Results page 8:”…is an extremely potent formula for authentic SARS-CoV-2 inhibition.”
- Discussion page 10:”…effectively block the spread of authentic SARS-CoV-2. ”
- Figure 4 legend page 12: “Inhibition of authentic SARS-CoV-2 infection by the 22 mAbs. Viral infected Vero E6 cells were identified by using nucleocapsid antibody (GeneTex GTX135357) conjugated to AF594 after cell fixation and permeabilization. Infected cells were quantified using Image J. (**c**). Representative images of cells infected with authentic SARS-CoV-2 in the presence of mAbs.
- Figure 6 legend page 14: “Inhibition of authentic SARS-CoV-2 infection…”
- Figure S8 legend page 26: “… infected with authentic SARS-CoV-2 following fixation and staining…”

*2. first part of the introduction could be improved*

We thank the reviewer for this comment, to improve the Introduction we made the following changes in the text (Page 2):

“

The COVID-19 pandemic is caused by the newly emerged SARS-Coronavirus-2 (SARS-CoV-2) and has incomparable public health, economic, and social consequences [1, 2]. Generation of neutralizing antibodies (nAbs) that specifically target the Receptor Binding Domain (RBD) of the SARS-CoV-2 Spike protein is thought to be essential in controlling SARS-CoV-2 [3-5].  Support of this assumption is the fact that RBD specific nAbs have been detected and isolated from convalescent patients that have recovered from COVID-19 as early as 8 days post diagnosis [6], exhibiting low clonal expansion and small numbers of somatic hyper mutations [7-10]. However, the critical survey of the humoral response to SARS-CoV-2 infection is somewhat confusing.  Some of the recoverees tend have robust and longlasting immunity while others display a waning of their neutralizing antibodies [6, 11].  What are the correlates of effective durable antibody response is still unclear.

“

*3. third paragraph of the results: the main text should explain how the RBD positive cells were identified*

We thank the reviewer for this comment. The following text was added to page 6

“

To investigate antibody responses in the Severe group, we used single B cell sorting using flow cytometry as previously described [7, 12] with SARS-CoV-2 RBD as a bait to derive monoclonal antibodies for analysis.

“

*4. Figure 7 is hard to interpret*

We thank the reviewer for this comment, and we apologize he found the presentation not clear. A **new version** of this figure is now incorporated, and we believe presents better the message, which is that the VJ combinations for neutralizing antibodies we isolated are frequent in the repertoire.

*5. Discussion: “Severe donors had higher level of shared B cell closed(?) compared to…”*

We thank the reviewer for reading carefully and we apologize for the misspell.

This has been corrected (page 9):

“Within the convalescent donors, Severe donors had higher level of shared B cell clones compared to Mild donors…”

*6. many have shown the effects of combination therapy (e.g. Regeneron antibodies) so the following should be modified or removed: “This is the first demonstration that combinations of neutralizing anti-SARS-CoV-2 nAbs can effectively block the spread of live SARS-CoV-2”*

We thank the reviewer for this comment. As the literature keeps changing, and new discoveries are being made on a daily basis, we are aware that we are not the first ones to show combinations of nAbs.

The following changes were made in the Discussion (Page 10):

“Combinations of nAbs were previously shown to effectively prevent the selection of HIV-1 escape mutants [13, 14]. While the mutation rates of SARS-CoV-2 are significantly lower compared to those of HIV-1, SARS-CoV-2 has been reported to mutate in culture in the presence of nAbs [15], emphasizing the importance of combination of antibodies targeting different weak spots on the virus. Recently, a combination of two nAbs targeting non-overlapping sites on SARS-CoV-2 Spike, effectively blocked the spread of SARS-CoV-2 in culture, as well as in a humanized mice model [16, 17]

“

**Reviewer #2:**

We thank the reviewer for pointing out that:” *This is a well-written manuscript describing the isolation and characterisation of SARS-CoV-2 neutralizing antibodies from infected individuals. The authors use the RBD to select B cells. Sequence analysis of SARS-CoV-2 specific mAbs are used to analyze the clonality of the immune response and are compared to those of “non-covid” individuals*” and for “*This is a timely study and adds to the body of research on Ab responses to SARS-CoV-2 infection*.”

Reviewer #2: Minor issues:

*1. The authors discuss pre-existing immunity to HCoV and cross-reactivity between SARS-CoV-2 in the first section of the results. However, they are using samples collected after SARS-CoV-2 infection so this section should be changed to take this into account.*

We thank the reviewer for this comment. Indeed, we have not tested plasma samples prior to SARS-CoV-2 infection, therefore we can only say with confidence that these antibody responses exist, not comment on when they appeared.

The text was changed accordingly:

“

Antibody responses against related respiratory viruses were previously reported to improve the outcomes of a SARS-CoV-2 infection[18]. “

*2. The authors isolate B cells binding to the RBD bait and analyze the sequences generated. However, in the downstream analysis it appears that not all the expressed mAbs bind to S. What do the authors think is the reason for this? If they are false positives in the sorting step then these sequences should not be included in the sequence analysis. This needs to be addressed in the text.*

We thank the reviewer for this question. Single cell sorting methods that isolate B cells through binding to fluorescently labeled, streptavidin conjugated baits not always bind soluble baits in solid phase assays such as ELISA. Onje example is the anti-HIV-1 antibody targeting the apex of the HIV-1 trimer BG01 [12]. This antibody was isolated with BG505 HIV-1 gp160 bait, and was extremely potent against the virus, yet did not bind the trimer in a simple ELISA. This can be due to the fact that the streptavidin conjugated tetrameric bait creates a different conformation, therefore exposing different epitopes that are recognized by the B cell in solution, but not by the antibodies in solid-phase assays. In the present study, mAb TAU-2212 does not bind to S protein, however it is the most potent neutralizing mAbs we have isolated. Therefore, binding to S, is not the only parameter for the mAb “being specific”, and we cannot exclude antibodies that do not bind S and call them “false positives”. Additionally, in other studies, using similar method, it has been shown as well that some of the antibodies that were sorted did not bind the cognate bait with which the B cells were sorted [7].

*3. It is not clear how the 9 and 13 mAbs from donors CoV01 and CoV02 were selected for further analysis. This should be clarified in the text.*

We thank the reviewer for this comment. To clarify this, we added the following sentence (Page 6):

“…(Next, we focused on antibodies that were part of the expanded clones as previously described [7, 12]. For sequences from donor CoV01, where we did not observe clonal expansion, we selected the most mutated sequences for production. We generated 9 and 13 monoclonal antibodies… “

*4. In figure 6b and 6c, the mAb alone (1109 or 2212) at 2ug/mL or 20ug/mL should be shown on the graph to compare with the combinations of mAbs and better show the synergistic effects*.

We thank the reviewer for this comment. Given the fact that we have proven clearly that mAbs have different specificities it would not make sense to adjust the total IgG concertation for the mAbs. However, to emphasize the benefit of using combinations of mAbs over high amounts of a single IgG we added to the graphs in Figure 6a (currently showing 1ug/ml nAbs), 10µg/ml of a single mAb for comparison. As the Reviewer can see, in most of the cases the triple combination, (that includes an overall concentration of 3µg/ml IgG) is more potent than the 10 µg/ml of a single mAb, proving that there is a clear effect when different antibodies are combined.

We added the following text (Page 9):

“

The nAbs we isolated are directed against at least three different sites on the virus; CR3022-proximal (TAU-1109), an unknown conformational epitope (TAU-2212) and the ACE2bs (TAU-2230, -2189, -1145, and -2303). Therefore, we combined nAbs TAU-2212 and TAU-1109 at a concentration of 1 and 10 µg/mL, with each one of the anti-ACE2bs nAbs, in a double and triple mixes (Figure 6). In general, nAb TAU-2212 exhibited a more substantial effect compared to nAb TAU-1109. Also, the combination of 1 µg/ml of three nAbs (TAU-1109 + TAU-2212 + ACE2bs nAb) was in all cases more potent than 10 µg/ml of a single ACE2bs nAb (with the exception of TAU-2230 where 10 µg/ml of the single nAb was as potent as 1 µg/ml of three nAbs, Figure 6a). When using 10 µg/ml of TAU-1109 and TAU-2212 in a triple combination with ACE2bs nAbs, complete viral inhibition was achieved in all cases, although most of the effect was contributed by TAU-2212 and not TAU-1109 (Figure 6b). We conclude that combining TAU-2212 with either ACE2bs nAbs or with TAU-1109 is a potent formula for authentic SARS-CoV-2 inhibition.

“

This is the new legend of the revised Figure 6 (pages 14-15)

“

**Figure 6: Combination of nAbs targeting different sites are more effective in SARS-CoV-2 neutralization.** Virus-induced cell death traced for 84 h when nAbs were combined with the virus 1 h prior to addition to confluent Vero E6 cells at MOI:1. The antibody concentration for each nAb is 1 µg/ml, and is depicted by circles (**a**), or 10 µg/ml and is depicted by squares (**b**). In every panel, the six nAbs are indicated on the top of each graph. In each graph, empty circles or squares correspond to the effect when the indicated nAb was used alone. Full circles or squares correspond to the effect when the indicated nAb was used in combination with TAU-1109 (depicted in purple), with TAU-2212 (depicted in orange), or both (depicted in red). The black bold line shows a “virus only” (no nAb) control.

“

*5. Is it not expected that the Ab response would be multi-clonal? Many other studies have shown that there are several neutralizing epitopes on S. These papers should be referenced in the discussion section.*

We thank the reviewer for this comment. We included the following recent references:

This was added to the Discussion (Page 10):

“

Similarly, to what has been reported [7, 19, 20], both donors, CoV01 and CoV02, exhibited neutralizing activity that was multi-clonal, *i.e.*, comprised of several neutralizing clones targeting different sites on the Spike.

“

*6. The authors highlight that some individuals with mild COVID-19 do not produce anti-RBD-IgG that could inhibit RBD:ACE2 interaction. Do these donors have neutralizing serum? If yes, would this not suggest that they produce nAbs targeting the other neutralizing epitopes identified?*

We thank the reviewer for this comment. While there are other epitopes on the surface of SARS-CoV-2 besides the RBD, and these epitopes could also drive neutralizing antibody responses in infected donors, in the past months it has been established in numerous studies [7, 16, 19-24] that the majority of the neutralizing antibody responses in infected donors is directed against the RBD. Therefore, our current study focused on the antibody responses to RBD as the primary target. We tested plasma binding in ELISA against RBD as well as the ability of the plasma to inhibit RBD:ACE2 binding and not neutralization where we could detect a significant difference between Mild and Severe donors. Our study does not come to exclude the possible presence of other plasma antibody responses that are directed against viral E and M proteins or other parts of the S protein outside the RBD.

To address the Reviewer’s concern, we added the following text:

Abstract (Page 2):

Severe and not mild infection correlated with high titers of IgG against Spike receptor binding domain (RBD) that were capable of ACE2:RBD inhibition.

Introduction (Page 4):

“

The Spike protein is responsible for viral attachment and entry into the host cells, and hence, is the target of most of the antibody responses [25-27]. Within the Spike protein, the receptor binding domain (RBD) is the minimal subunit that mediates viral entry [4, 5] and was reported to be the main target for neutralizing antibodies elicited during infection [7, 16, 19-24, 28].

”

And Discussion (Page 10):

“

In the present study we analyzed the B cell responses in convalescent COVID-19 donors, while focusing on antibodies produced in two donors with severe disease manifestations. By now, several studies showed that COVID-19 severity correlates with neutralization[7, 11, 29], and that anti-RBD antibody response dominates SARS-CoV-2 neutralization, although neutralizing antibodies can be directed against other viral determinants besides the RBD [9, 19].

“

*7. In the final paragraph of the discussion, the authors conclude that this is the first study to show combinations of mAbs can block spread of live SARS-CoV-2. However, the data for a mAb on its own is not shown for comparison. The relevance of this observation should be included within the discussion. Why is the spreading assay more relevant than a neutralization assay for example? Other labs have also tried combinations and these should be referenced/discussed.*

We thank the Reviewer for this important point. To make the figure clearer, Figure 6 was changed, now presenting the effect of every mAb used alone and in combination in two different concentrations – 1 µg/ml and 10 µg/ml. This is the revised legend of Figure 6 (Page 14):

**“**

**Figure 6: Combination of nAbs targeting different sites are more effective in SARS-CoV-2 neutralization.** Virus-induced cell death traced for 84 h when nAbs were combined with the virus 1 h prior to addition to confluent Vero E6 cells at MOI:1. The antibody concentration for each nAb is 1 µg/ml, and is depicted by circles (**a**), or 10 µg/ml and is depicted by squares (**b**). In every panel, the six nAbs are indicated on the top of each graph. In each graph, empty circles or squares correspond to the effect when the indicated nAb was used alone. Full circles or squares correspond to the effect when the indicated nAb was used in combination with TAU-1109 (depicted in purple), with TAU-2212 (depicted in orange), or both (depicted in red). The black bold line shows a “virus only” (no nAb) control. “

As to Reviewer’s question about the relevance of the spreading assay *versus* neutralization assay, there is no superiority of one assay over the other, however while looking on the % infection requires fixing the cells, which ultimately means that they can be examined in one timepoint, the main advantage of the spreading assay is the ability to track the culture over time, since what is assayed is the dead cells number.

As to Reviewer’s comment about other labs trying combinations of mAbs, unfortunately this was published during the submission process, therefore we are aware that we are not the first ones to show combinations of nAbs, therefore the following changes were introduced in the text in the Discussion section (Page 11):

“Combinations of nAbs were previously shown to effectively prevent the selection of HIV-1 escape mutants [13, 14]. While the mutation rates of SARS-CoV-2 are significantly lower compared to those of HIV-1, SARS-CoV-2 has been reported to mutate in culture in the presence of nAbs [15], emphasizing the importance of combination of antibodies targeting different weak spots on the virus. Recently, a combination of two nAbs targeting non-overlapping sites on SARS-CoV-2 Spike, effectively blocked the spread of SARS-CoV-2 in culture, as well as in a humanized mice model [16, 17].

“

*8. More references are required to other studies on SARS-CoV-2 Ab responses are needed. In particular the part referring to the relationship between disease severity and magnitude of the Ab response.*

We thank the reviewer for this comment. We added the following recent references that were also addressed in the text:

3. Klasse, P. J. & Moore, J. P. Antibodies to SARS-CoV-2 and their potential for therapeutic passive immunization. *Elife* **9**, doi:10.7554/eLife.57877 (2020).

5. Tai, W. *et al.* Characterization of the receptor-binding domain (RBD) of 2019 novel coronavirus: implication for development of RBD protein as a viral attachment inhibitor and vaccine. *Cell Mol Immunol* **17**, 613-620, doi:10.1038/s41423-020-0400-4 (2020).

9. Liu, L. *et al.* Potent neutralizing antibodies against multiple epitopes on SARS-CoV-2 spike. *Nature* **584**, 450-456, doi:10.1038/s41586-020-2571-7 (2020).

10. Ju, B. *et al.* Human neutralizing antibodies elicited by SARS-CoV-2 infection. *Nature* **584**, 115-119, doi:10.1038/s41586-020-2380-z (2020).

11. Seow, J. *et al.* Longitudinal observation and decline of neutralizing antibody responses in the three months following SARS-CoV-2 infection in humans. *Nat Microbiol*, doi:10.1038/s41564-020-00813-8 (2020).

14. Pillay, T. S. Gene of the month: the 2019-nCoV/SARS-CoV-2 novel coronavirus spike protein. *J Clin Pathol* **73**, 366-369, doi:10.1136/jclinpath-2020-206658 (2020).

15. Sui, J. *et al.* Potent neutralization of severe acute respiratory syndrome (SARS) coronavirus by a human mAb to S1 protein that blocks receptor association. *Proc Natl Acad Sci U S A* **101**, 2536-2541, doi:10.1073/pnas.0307140101 (2004).

16. Wang, Q. *et al.* Structural and Functional Basis of SARS-CoV-2 Entry by Using Human ACE2. *Cell*, doi:10.1016/j.cell.2020.03.045 (2020).

17. Baum, A. *et al.* Antibody cocktail to SARS-CoV-2 spike protein prevents rapid mutational escape seen with individual antibodies. *Science* **369**, 1014-1018, doi:10.1126/science.abd0831 (2020).

20. Huo, J. *et al.* Neutralizing nanobodies bind SARS-CoV-2 spike RBD and block interaction with ACE2. *Nat Struct Mol Biol*, doi:10.1038/s41594-020-0469-6 (2020).

21. Jiang, S., Hillyer, C. & Du, L. Neutralizing Antibodies against SARS-CoV-2 and Other Human Coronaviruses. *Trends Immunol* **41**, 355-359, doi:10.1016/j.it.2020.03.007 (2020).

22. Liu, L. *et al.* Potent Neutralizing Monoclonal Antibodies Directed to Multiple Epitopes on the SARS-CoV-2 Spike. *bioRxiv*, doi:10.1101/2020.06.17.153486 (2020).

24. Piccoli, L. *et al.* Mapping Neutralizing and Immunodominant Sites on the SARS-CoV-2 Spike Receptor-Binding Domain by Structure-Guided High-Resolution Serology. *Cell*, doi:10.1016/j.cell.2020.09.037 (2020).

35. Lee, N. *et al.* Anti-SARS-CoV IgG response in relation to disease severity of severe acute respiratory syndrome. *J Clin Virol* **35**, 179-184, doi:10.1016/j.jcv.2005.07.005 (2006).

41. Hansen, J. *et al.* Studies in humanized mice and convalescent humans yield a SARS-CoV-2 antibody cocktail. *Science* **369**, 1010-1014, doi:10.1126/science.abd0827 (2020).

*9. Page 7: what was the potency of the live virus neutalization compared to the pseudovirus?*

We thank the reviewer for this comment. We included the following text to clarify this point (Page 7):

“

Five of the nine mAbs that neutralized the pseudo-typed virus, also neutralized authentic virus when used at concentration 10 µg/mL (TAU-1109, -2212, -2189, -2230, and -2303, Figure 4b and c, Supplementary Figure 10), while four mAbs that demonstrated neutralizing activity in the pseudo-viral assay (TAU-1115, -1145, -2220 and -2310) did not inhibit authentic SARS-CoV-2.

“

*10. Page 7: TAU-2212 does not bind S in ELISA but does on the cell surface and has neutralizing activity. The authors suggest it may be binding an epitope involving more than one subunit. The authors do not state what form the S is that is used in ELISA. Is it a stabilised trimer?*

We used a stabilized Spike trimer, similar to the one used in [30]. The following text was added (Page 6)

“….bound SARS CoV-2 RBD and to stabilized Spike trimer [30] protein by ELISA…”

*11. Page 9: The data presented does not justify the conclusion that “these nAbs can be produced by the majority of individuals”. “may” would be more appropriate.*

We thank the reviewer for this comment. The text was changed accordingly (Page 9)

“Therefore, given enough antigenic stimulation (which is the case during severe COVID-19), these nAbs may be produced by the majority of individuals (Figure 7).”

--END--

References:

1.	Zhou, P., et al., *A pneumonia outbreak associated with a new coronavirus of probable bat origin.* Nature, 2020. **579**(7798): p. 270-273.

2.	Wu, F., et al., *A new coronavirus associated with human respiratory disease in China.* Nature, 2020. **579**(7798): p. 265-269.

3.	Klasse, P.J. and J.P. Moore, *Antibodies to SARS-CoV-2 and their potential for therapeutic passive immunization.* Elife, 2020. **9**.

4.	Lan, J., et al., *Structure of the SARS-CoV-2 spike receptor-binding domain bound to the ACE2 receptor.* Nature, 2020.

5.	Tai, W., et al., *Characterization of the receptor-binding domain (RBD) of 2019 novel coronavirus: implication for development of RBD protein as a viral attachment inhibitor and vaccine.* Cell Mol Immunol, 2020. **17**(6): p. 613-620.

6.	Kreer, C., et al., *Longitudinal Isolation of Potent Near-Germline SARS-CoV-2-Neutralizing Antibodies from COVID-19 Patients.* Cell, 2020. **182**(4): p. 843-854 e12.

7.	Robbiani, D.F., et al., *Convergent antibody responses to SARS-CoV-2 in convalescent individuals.* Nature, 2020.

8.	Wec, A.Z., et al., *Broad neutralization of SARS-related viruses by human monoclonal antibodies.* Science, 2020.

9.	Liu, L., et al., *Potent neutralizing antibodies against multiple epitopes on SARS-CoV-2 spike.* Nature, 2020. **584**(7821): p. 450-456.

10.	Ju, B., et al., *Human neutralizing antibodies elicited by SARS-CoV-2 infection.* Nature, 2020. **584**(7819): p. 115-119.

11.	Seow, J., et al., *Longitudinal observation and decline of neutralizing antibody responses in the three months following SARS-CoV-2 infection in humans.* Nat Microbiol, 2020.

12.	Freund, N.T., et al., *Coexistence of potent HIV-1 broadly neutralizing antibodies and antibody-sensitive viruses in a viremic controller.* Sci Transl Med, 2017. **9**(373).

13.	Klein, F., et al., *HIV therapy by a combination of broadly neutralizing antibodies in humanized mice.* Nature, 2012. **492**(7427): p. 118-22.

14.	Mendoza, P., et al., *Combination therapy with anti-HIV-1 antibodies maintains viral suppression.* Nature, 2018. **561**(7724): p. 479-484.

15.	Weisblum, Y., et al., *Escape from neutralizing antibodies by SARS-CoV-2 spike protein variants.* bioRxiv, 2020.

16.	Baum, A., et al., *Antibody cocktail to SARS-CoV-2 spike protein prevents rapid mutational escape seen with individual antibodies.* Science, 2020. **369**(6506): p. 1014-1018.

17.	Hansen, J., et al., *Studies in humanized mice and convalescent humans yield a SARS-CoV-2 antibody cocktail.* Science, 2020. **369**(6506): p. 1010-1014.

18.	Lynch, R.M., et al., *Augmented Zika and Dengue Neutralizing Antibodies Are Associated With Guillain-Barre Syndrome.* J Infect Dis, 2019. **219**(1): p. 26-30.

19.	Brouwer, P.J.M., et al., *Potent neutralizing antibodies from COVID-19 patients define multiple targets of vulnerability.* Science, 2020.

20.	Liu, L., et al., *Potent Neutralizing Monoclonal Antibodies Directed to Multiple Epitopes on the SARS-CoV-2 Spike.* bioRxiv, 2020.

21.	Cao, Y., et al., *Potent Neutralizing Antibodies against SARS-CoV-2 Identified by High-Throughput Single-Cell Sequencing of Convalescent Patients' B Cells.* Cell, 2020. **182**(1): p. 73-84 e16.

22.	Huo, J., et al., *Neutralizing nanobodies bind SARS-CoV-2 spike RBD and block interaction with ACE2.* Nat Struct Mol Biol, 2020.

23.	Jiang, S., C. Hillyer, and L. Du, *Neutralizing Antibodies against SARS-CoV-2 and Other Human Coronaviruses.* Trends Immunol, 2020. **41**(5): p. 355-359.

24.	Rogers, T.F., et al., *Isolation of potent SARS-CoV-2 neutralizing antibodies and protection from disease in a small animal model.* Science, 2020.

25.	Pillay, T.S., *Gene of the month: the 2019-nCoV/SARS-CoV-2 novel coronavirus spike protein.* J Clin Pathol, 2020. **73**(7): p. 366-369.

26.	Sui, J., et al., *Potent neutralization of severe acute respiratory syndrome (SARS) coronavirus by a human mAb to S1 protein that blocks receptor association.* Proc Natl Acad Sci U S A, 2004. **101**(8): p. 2536-41.

27.	Wang, Q., et al., *Structural and Functional Basis of SARS-CoV-2 Entry by Using Human ACE2.* Cell, 2020.

28.	Piccoli, L., et al., *Mapping Neutralizing and Immunodominant Sites on the SARS-CoV-2 Spike Receptor-Binding Domain by Structure-Guided High-Resolution Serology.* Cell, 2020.

29.	Lee, N., et al., *Anti-SARS-CoV IgG response in relation to disease severity of severe acute respiratory syndrome.* J Clin Virol, 2006. **35**(2): p. 179-84.

30.	Amanat, F., et al., *A serological assay to detect SARS-CoV-2 seroconversion in humans.* Nat Med, 2020. **26**(7): p. 1033-1036.
